# High-risk human papillomavirus genotype distribution among women living with HIV; implication for cervical cancer prevention in a resource limited setting

**DOI:** 10.1186/s13027-023-00513-y

**Published:** 2023-05-26

**Authors:** Patrick Kafui Akakpo, Sebastian Ken-Amoah, Nancy Innocentia Ebu Enyan, Elizabeth Agyare, Emmanuel Salia, Ibrahim Baidoo, Leonard Derkyi-Kwarteng, Matthew Asare, George Adjei, Stephen Ayisi Addo, Dorcas Obiri-Yeboah

**Affiliations:** 1 Department of Anatomic Pathology, School of Medical Sciences, University of Cape Coast, Cape Coast, Ghana; 2 Department of Obstetrics and Gynaecology, School of Medical Sciences, University of Cape Coast, Cape Coast, Ghana; 3 Department of Adult Health, School of Nursing and Midwifery, University of Cape Coast, Cape Coast, Ghana; 4 Public Health Unit, Cape Coast Teaching Hospital, Cape Coast, Ghana; 5 Department of Microbiology and Immunology, School of Medical Sciences, University of Cape Coast, Cape Coast, Ghana; 6 Department of Public Health, Robbins College of Health and Human Services, Baylor University, Waco, TX USA; 7 Department of Community Medicine, School of Medical Sciences, University of Cape Coast, Cape Coast, Ghana; 8 National AIDS/STIs Control Programme, Korle-Bu, Accra, Ghana

**Keywords:** HPV, HIV, Ghana, Cervical cancer, Screening, Resource-limited settings

## Abstract

**Background:**

For women living with HIV (WLHIV), the burden of persistent HPV infection, cervical pre-cancerous lesions and cancer have been demonstrated to be higher than among HIV-negative women. As Ghana and other lower-middle-income countries (LMIC) work toward developing national cervical cancer programmes, it is essential that local scientific evidence be provided to guide policy decisions, especially for such special populations. The objective of this study was to determine the distribution of high-risk HPV genotype and related factors among WLHIV and its implication for the prevention of cervical cancer prevention efforts.

**Methods:**

A cross-sectional study was conducted at the Cape Coast Teaching Hospital in Ghana. WLHIV, aged 25–65 years, who met the eligibility criteria were recruited through a simple random sampling method. An interviewer-administered questionnaire was used to gather socio-demographic, behavioural, clinical and other pertinent information. The AmpFire HPV detection system (Atila BioSystem, Mointain View, CA was used to detect 15 high-risk HPV genotypes from self-collected cervico-vaginal samples. The data collected were exported to STATA 16.0 for statistical analysis.

**Results:**

In all, 330 study participants, with mean age of 47.2 years (SD ± 10.7), were involved. Most (69.1%, n = 188/272) had HIV viral loads < 1000 copies/ml and 41.2% (n = 136) had ever heard of cervical screening. The overall hr-HPV prevalence was 42.7% (n = 141, 95% CI 37.4–48.1) and the five commonest hr-HPV types among screen positives were HPV59 (50.4%), HPV18 (30.5%), HPV35 (26.2%), HPV58 (17%) and HPV45 (14.9%). Most infected women (60.3%, n = 85) had multiple hr-HPV infections, with about 57.4% (n = 81) having 2–5 h-HPV types, while 2.8% (n = 4) had more than five hr-HPV types. A total of 37.6% (n = 53) had HPV16 and/or18, while 66.0% (n = 93) had the hr-HPV genotypes covered by the nonavalent vaccine. Women with HIV viral load ≥ 1000copies/ml (AOR = 5.58, 95% CI 2.89–10.78, *p* < 0.001) had a higher likelihood of being co-infected.

**Conclusion:**

This study found out that the prevalence of hr-HPV still remains high in women with HIV, with a notable occurrence of multiple infections and infection with genotypes 16 and/or18. Additionally, an association was established between hr-HPV and infection HIV viral load.. Therefore, comprehensive HIV care for these women should include awareness of cervical cancer, consideration of vaccination and implementation of screening and follow-up protocols. National programmes in LMIC, such as Ghana, should consider using HPV-based screen-triage-treat approach with partial genotyping.

## Background

Cervical Cancer remains a major public health problem globally, with a global incidence rate of 3.1 per 100,000 women [1, 2] in 2018. The global age-standardised incidence rate was 13.3 per 100,000 in 2020 [3]. It is known to disproportionately affect women in developing countries, where most cases and mortality are reported [3, 4]. The evidence suggests an increasing trend of incidence in sub-Saharan Africa [5]. In Western Africa, the incidence was 23.0 per 100,000 women, with a mortality rate of 16.6 per 100,000 women in 2020 [6]. In Ghana, it is estimated that 2797 women develop cervical cancer annually, with 1699 deaths and a crude incidence rate of 18.3 per 100,000 population [7].

The main aetiologic factor, persistent infection with sexually transmitted high-risk Human papillomavirus (hr-HPV), has been identified and the contribution of various genotypes has been a focus of several studies in different geographical locations [8, 9]. HPV can be classified into high-risk and low-risk. Low-risk HPV mainly causes genital warts or may cause no disease, while high-risk HPVs can cause several types of cancers, including cervical cancer. There are about 14 high-risk HPV types. They include HPV 16, 18, 31, 33, 35, 39, 45, 51, 52, 56, 58, 59, 66, and 68 [10]. Two of these genotypes, HPV16 and HPV 18, are reported to be responsible for most HPV-related cancers [11]. It is important to note that the distribution of HPV genotypes may vary across different geographical regions of the world. In Ghana, for example, HPV 35 has been reported to be a common circulating genotype [12]. The causal role of different genotypes, in relation to cervical pre-cancer may also vary and therefore, differ, depending on the region. Among women with normal cytology, it is 3.8%, compared with 24.9% in low-grade cervical lesions and 38.6% in high-grade cervical abnormalities [7]. Studies have reported a high prevalence of hr-HPV genotypes 18, 59 and 45 among women with cervical cancer who were seeking treatment at a major teaching hospital in Ghana [13]. Similarly, the prevalence of single hr-HPV prevalence of 32.4% and 9.7% for several high-risk types was recorded in the North Tongu district of Ghana [14]. Apart from HPV, other risk factors for cervical cancer include HIV/AIDS, early-age onset of sexual activities; a high number of sexual partners, long-term use of oral contraceptives; and other hormonal influences [15].

Studies have shown that the burden of cervical pre-cancerous lesions and cancer for Women living with HIV (WLHIV), is higher, compared to HIV-negative women [4–6] WLHIV are more likely to have persistent HPV infection, which can lead to the development of cervical intraepithelial neoplasia (CIN) and invasive cervical carcinoma (ICC) [12], as compared to women, without HIV, due to their immunosuppressive state [12, 16, 17]. This brings to light the importance of managing WLHIV well, with and persistent use of anti-retroviral medications to increase their immunity, leading to increased clearance of hr-HPV and thus, reducing the incidence of cervical cancer in this population [18–20].

A study conducted in Europe found that hr-HPV screening reduced the incidence of invasive cervical cancer (ICC) by about 60–70%, compared with cytology and allows for longer screening intervals [21]. Such findings have informed screening algorithms in many counties.

Globally, there is a gap in specific protocols for cervical cancer prevention for WLHIV. Questions remain on the best approach, in terms of the age at which to begin screening, the possible role of HPV vaccination, frequency of screening, the screening tests and many more. These questions arise as the evidence accumulated from studies among WLHIV shows that the epidemiology of HPV and cervical cancer is definitely modified by co-infection with HIV [22, 23] and the impact of anti-retroviral therapy (ART) on this epidemiology [24–26]. This calls for specific attention for this sub-group of the population.

Efforts to eliminate cervical cancer require a combination of approaches that includes awareness creation, implementing screening measures and vaccination. Apart from studies that have investigated the prevalence of HPV among Ghanaians, other studies have focused on the awareness and knowledge of women with and without HIV [27, 28]. Generally, knowledge of HPV and cervical cancer among WLHIV in Ghana was good, although it was observed to be lower in women with lower socioeconomic status [29–31]. Despite this, there is still a low uptake of cervical screening services [27, 31, 32], indicating a need for improved access and utilization of such services through well-designed educational programmes.

As Ghana, through the Ghana Health Service (GHS), works toward developing a cervical cancer programme, it is essential that scientific evidence be provided to guide policy decisions on women, particularly high-risk populations. The siloed approach to HIV, HPV and cervical cancer research has not been very impactful, hence there is a need for a more comprehensive and multidisciplinary approach, which will offer a more nuanced understanding and showcase how progress can be sustainable. This study aimed at determining the distribution of high-risk HPV genotype and related factors among WHIV and its implication for the prevention of cervical cancer in a LMIC such as Ghana.

## Materials and methods

### Study design and site

A hospital-based analytical cross-sectional study was conducted at the HIV clinic of Cape Coast Teaching Hospital (CCTH), in Ghana fromNovember, 2020 to April, 2021. CCTHis a tertiary hospital, which serves the Central and the Western regions of Ghana and well as the south-western part of the country. It is a 400-bed capacity hospital and provides specialist care across more than 20 departments and units, covering a range of medical and surgical specialties. It has a well-structured HIV/AIDS clinic that operates every week of the year. The facility has the capacity to offer a wide range of services for the prevention, screening and management of cervical cancer, but unfortunately, these services have remained largely uncoordinated and opportunistic, which is reflective of the national situation.

### Study population and sampling

Women living with HIV (WLHIV), who were registered to receive care at the HIV clinic constituted the study population. Eligible participants were WLHIV between the ages of 25 and 65 yearswith the exclusion of those, who were pregnant, had undergone total hysterectomy, local treatment for cervical lesions or had never engaged in peno-vaginal sexual intercourse.

The prevalence of hr-HPV among WLHIV aged ≥ 18 years, in Cape Coast was 60.6% [12] and based on the data at the ART clinic, the total adult population was 2,106. With a 95% confidence level, 5% margin of error and 5% provision for contingency the estimated sample size was 329. A simple random sampling method was used in selecting study participants. Women were asked to pick from a box with papers having a “yes” or a “no” written on them during each clinic day. Eligible WLHIV who were menstruating at the time of recruitment had the option to rebook their sampling collection date. The clinic has an average of 120 clients attending with about 65% being adult females. The recruitment strategy ensured that not more than 25 eligible women were recruited per week to ensure the spread of opportunity among clients. Written informed consent was obtained from all participants before recruitment.

#### Data collection

The study participants were interviewed by trained research assistants (community health and public health nurses) using a pre-tested structured questionnaire that included their socio-demographic characteristics, their knowledge of HPV and cervical cancer, their reproductive health and other characteristics. Additional clinical characteristics relating to the HIV care, including viral load, were obtained from the HIV clinic records. The women were then trained to take their cervico-vaginal samples by themselves using a brush. The specimen was then placed in a labelled tube, sealed, and transported to the testing laboratory within 48 h without any transport medium. A screen, triage and treat approach, as recommended by WHO, was followed.

HPV DNA testing with genotyping was used as the primary screening test for all participants. All screen-negatives were counselled to rescreen with HPV DNA test in 3 years. Screen-positives were stratified into 2 groups based on the genotypes of the high-risk HPV found: those with HPV genotype 16 or 18 and those with other high-risk HPV. Those in the first group were immediately assessed, after application of acetic acid 5% on the cervix, for eligibility for ablative therapy, based on the visibility and position of the transformation zone and the location and size of the lesion, if the patient has a visible lesion. Those eligible for ablation were treated with thermocoagulation, using the Liger Medical HTU-110 ThermoCoagulator. Those ineligible for ablation, received loop electrosurgical excision procedure (LEEP), using the Liger Medical ESU-110 electrosurgical generator, and the specimen sent for histopathology evaluation. If the histopathology evaluation indicates a high-grade lesion, adenocarcinoma in situ (AIS) or cancer, the client is referred for further evaluation and treatment. If the histopathology evaluation indicates a low-grade lesion, such clients, as well as those who received ablative treatment will be reevaluated in one year.

The screen-positives for other high-risk HPV were triaged, using visual inspection with acetic acid (VIA), if a client is 45 years old or younger, and cytology (Pap test), if the client is older than 45 years. This segregation was based on the changes in location of the transformation zone with age. All VIA and cytology screen-negatives were counselled to repeat the HPV test in one year. If they tested negative again, they move routine interval screening schedule. Should a client test positive or found to have a lesion on the cervix, suspicious of cancer on VIA, tests positive for a high-grade lesion on Pap test or tests positive again for other high-risk HPV on repeat screening, she undergoes colposcopy and biopsy and the specimen sent for histopathology evaluation. Subsequently, she undergoes further evaluation and treatment, depending on the findings of the colposcopy and histopathology report.

#### Hr-HPV detection and genotyping

AmpFire HPV detection system (Atila BioSystems, Mountain View, CA) was used. It is an isothermal PCR assay that individually detects15 high risk HPV [16, 29, 31, 33–44] types [34, 45]. The AmpFire full HPV genotyping kit targets the L1, E6 and E7 genes to optimize the detection and contains the Reaction Mix (with buffer, enzymes, and dNTPs), Primer Mix (with primers and probes), positive control, and negative control. The samples were tested with strict adherence to the manufacturers protocol. Negative Control and Positive Control are included in each assay to ensure the quality of the assay performance and rule out contamination. Only samples where both positive and negative controls passed were taken as valid results. About 30 samples were repeated to ensure the reproducibility of the results.

### Data management and statistical analysis

All data for this study were entered using a Computer-Assisted Personal Interview (CAPI) developed by the Centre for Data Archiving, Management, Analysis and Advocacy (C-DAMAA) at the Department of Economics, University of Cape Coast. This ensured that all the controls were in place to ensure data quality and the data is hosted on a secure server and accessible only to the assigned administrator. Data were, subsequently received from the administrator and exported to STATA 16.0 for statistical analysis.

All categorical sociodemographic variables were described using frequencies and percentages. Means and corresponding standard deviations were used to describe sociodemographic variables that were continuous and normally distributed. High-risk HPV prevalence and genotypes among HIV/HPV co-infected women were analysed using frequencies, percentages, and charts.

Seventeen questions were used to assess participants’ knowledge of cervical cancer. Each correct response to a question was scored one [1] and zero (0) otherwise. The total score for each participant for all the seventeen questions was calculated. The distribution of the total scores for all the participants was found to be skewed with a median of 4.5. Therefore, participants with total scores less than or equal to 4.5 were classified as having insufficient knowledge whereas those with total scores greater than 4.5 were classified as having sufficient knowledge.

Chi-square tests were used to examine the association between HIV/HPV co-infection status of study participants and socio-demographic, knowledge and behavioural variables. All variables with *p* values < 0.1 were used to construct both bivariate and multivariable logistic regressions with HIV/HPV status as the dependent variable. Age and viral load were considered as a priori variables. Hence, irrespective of whether *p* values of age and viral load were less than 0.1, they were considered in both the bivariate and multivariable logistic regressions. Similar method was used to construct another bivariate and multivariate logistic regressions with HPV 16 and/or 18 genotype status of study participants as dependent variable and socio-demographic, knowledge and behavioural variables as independent variables.

Bivariate and multivariable regression with the main outcome as women’s cervical cancer Knowledge (insufficient or sufficient knowledge) were also performed. Chi-squared tests were initially performed to examine the association between cervical cancer knowledge and women’s sociodemographic and behavioural characteristics. The variables that were associated using the chi-squared tests were used to construct both the bivariate and multivariable logistic regressions. All statistical tests were two-sided and *p* values < 0.05 were considered statistically significant.

## Results

### Socio-demographic, behavioral, and clinical characteristics of participants

**Fig. 1:**
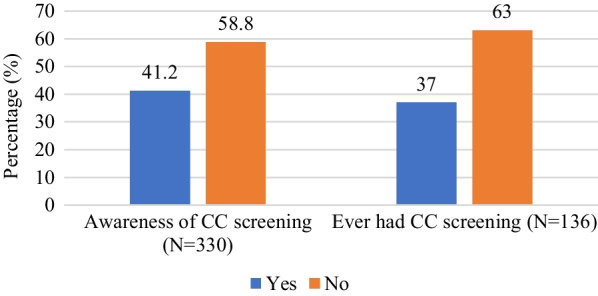
Awareness andexperience with CC screening among study participants

**Fig. 2:**
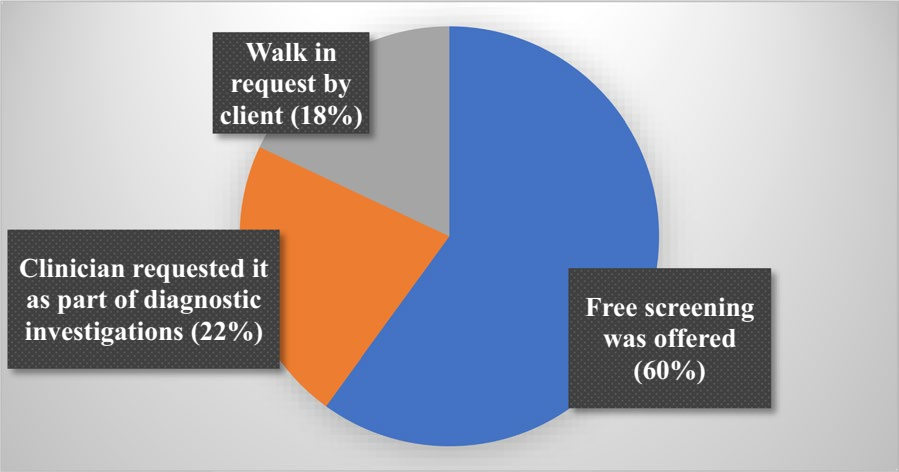
Reasons for previous screening among 50 WLHIV who had ever had cervical screening (N = 50)

A total of 330 study participants’ results are presented. The mean age was 47.2 years (SD ± 10.7). Most of the participants have current partners (37.6%) and 57.9% are involved in unskilled work. The median number of pregnancies was 4 (IQR 2–5) and most (51.2%) had 3–6 lifetime sexual partners and the mean age at first sex was 18.7 years (SD ± 3.3). Among those who had been sexually active in the past 3 months (N = 172) consistent condom usage rate was 77.9%. Most (69.1%) of the women had their HIV viral loads < 1000 copies/ml as against 30.9% whose viral loads were ≥ 1000 copies/ml. See Table 1. A total of 41.2% had ever heard of cervical screening of which only 37% had ever had screening mainly due to an offer of free screening (Figs. 1, 2).

**Table 1:** Socio-demographic, behavioral and clinical characteristics of study participants (N = 330)

Variables	Number (n)	Percentage (%)
Age (years)		
Mean, SD	47.2	10.7
25–34	40	12.1
35–44	95	28.8
45–54	107	32.4
≥ 55	88	26.7
Educational level		
No formal education/primary	137	41.5
JHS/Secondary	164	49.7
Tertiary	29	8.8
Marital status		
Single	75	22.7
Married/cohabiting	124	37.6
Widowed/divorced	131	39.7
Occupation		
Unemployed	68	20.6
Unskilled work	191	57.9
Skilled work	71	21.5
Religion		
Christianity	296	89.7
Islam	34	10.3
Number of pregnancies		
Median, IQR	4	2–5
0	19	5.76
1–3	135	40.91
4–6	134	40.60
≥ 7	42	12.72
Number of children		
Median, IQR	3	2–4
0	35	10.7
1–3	176	53.3
4–6	105	31.8
≥ 7	14	4.2
Number of Lifetime Sexual partners (N = 328)		
Median, IQR	3	2–3
1–2	157	47.9
3–6	168	51.2
≥ 7	3	0.9
Age at first sex (326)		
Mean, SD	18.7	3.3
≤ 16	95	28.1
17–25	221	67.8
≥ 26	10	3.03
Currently sexually active (last 3 months)		
Yes	182	55.2
No	148	44.8
Condom use in last 3 months (N = 172)		
Yes	134	77.9
No	38	22.1
Ever used hormonal contraceptives		
Yes	129	39.1
No	201	60.9
Current hormonal contraceptive use		
Yes	53	16.1
No	277	83.9
Menarche (years) (N = 325)		
Mean, SD	15.3	2.0
≤ 13	47	14.5
14–19	267	82.2
≥ 20	11	3.3
Still menstruates		
Yes	153	46.4
No	177	53.6
Currently smokes cigarettes		
Yes	6	1.8
No	324	98.2
Duration of HIV Diagnosis (years)		
Median, IQR	4.0	1.3–8.0
< 1	40	12.1
1–4	142	43.0
5–10	98	29.7
> 10	50	15.2
Duration on ART (years)		
Median	46	15–80
< 1	52	15.8
1- < 5	160	48.5
5–10	75	22.7
> 10	43	13.0
HIV viral load (copies/ml) (N = 272)		
Median	411.5	36–1588
Target not detected	34	12.5
< 20	0	0.0
20–999	154	56.6
≥ 1000	84	30.9

### Prevalence of hr-HPV and genotypes distribution among study participants

**Fig. 3:**
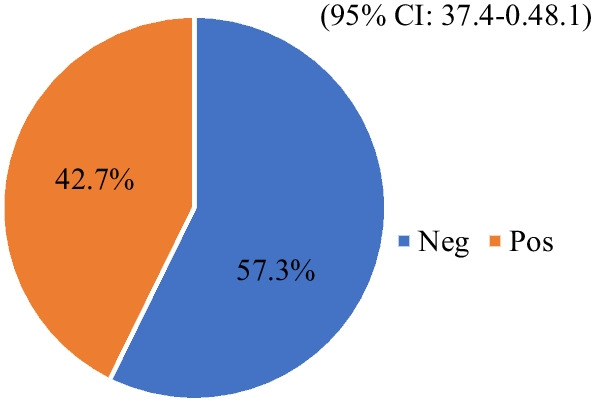
The distribution of hr-HPV positive and hr-HPV negative among study participants (N = 330)

**Fig. 4:**
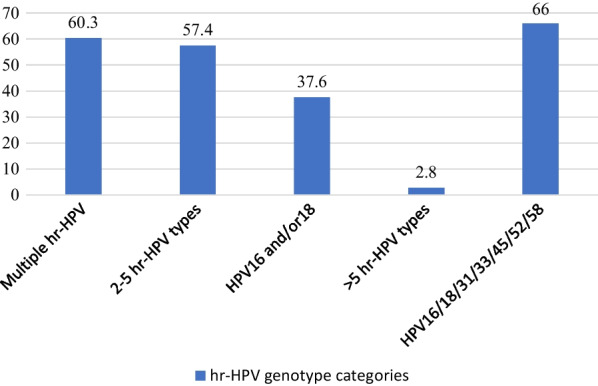
Types of hr-HPV genotype categories of coinfected participants (N = 141)

The overall hr-HPV prevalence was 42.7% (N = 141, 95% CI 37.4–48.1) of the study population (Fig. 3). The top five most prevalent hr-HPV types were found to be HPV59 (50.4%), HPV18 (30.5%), HPV35 (26.2%), HPV58 (17.0%) and HPV45 (14.9%) (Table 2). Most (60.3%) of co-infected women had multiple hr-HPV infections, 57.4% with 2–5 h-HPV types whilst 2.8% had more than five [5] hr-HPV types. A total of 37.6% had HPV16 and/or 18 while 66.0% had the hr-HPV genotypes covered by the nonavalent vaccine (Fig. 4).

**Table 2:** HPV prevalence and distribution of HPV genotypes among HIV/HPV co-infected women (N = 141)

Type of HPV genotype	Number of HPV	Percentage (%)
HPV16	13	9.2
HPV18	43	30.5
HPV31	16	11.3
HPV33	4	2.8
HPV35	37	26.2
HPV39	10	7.1
HPV45	21	14.9
HPV51	9	6.4
HPV52	20	14.2
HPV53	19	13.5
HPV56	12	8.5
HPV58	24	17.0
HPV59	71	50.3
HPV66	13	9.2
HPV68	17	12.1

### Predictors of HIV/HPV co-infection

Table 3 depicts both bivariate and multivariable regressions with HIV/HPV co-infection status as the main dependent variable. In the multivariable regression, women who had not heard of cervical cancer screening (AOR = 2.80, 95% CI 1.46–5.35, *p* = 0.002) and those with HIV viral load ≥ 1000 copies/ml (AOR = 5.58, 95% CI 2.89–10.78, *p* < 0.001) had a higher likelihood of being co-infected.

**Table 3:** Predictors of HIV/HPV co-infection

Variable	HIV/HPV co-infection		OR (95% CI)	*P* value	AOR (95% CI)	*P* value
	Positive	Total				
	n (%)	N (%)				
Age (years)*						
25–34	20 (50.0)	40 (100.0)	1		1	
35–44	43 (45.3)	95 (100.0)	0.83 (0.39–1.73)	0.615	0.43 (0.16–1.16)	0.096
45–54	47 (43.9)	107 (100.0)	0.78 (0.38–1.62)	0.511	0.50 (0.18–1.34)	0.168
≥ 55	31 (35.2)	88 (100.0)	0.54 (0.25–1.16)	0.116	0.23 (0.07–0.71)	0.011
Marital status						
Single	39 (52.0)	75 (100.0)	1		1	
Married/cohabiting	45 (36.3)	124 (100.0)	0.53 (0.29–0.94)	0.031	0.67 (0.32–1.39)	0.278
Widowed/divorced	57 (43.5)	131 (100.0)	0.71 (0.40–1.26)	0.241	1.14 (0.52–2.48)	0.739
Heard of cervical cancer screening?						
Yes	50 (36.8)	136 (100.0)	1		1	
No	91 (46.9)	194 (100.0)	1.52 (0.97–2.38)	0.067	2.80 (1.46–5.35)	0.002
Duration on ART						
< 1	24 (46.2)	52 (100.0)	1		1	
1–4	57 (35.6)	160 (100.0)	0.65 (0.34–1.22)	0.176	0.32 (0.08–1.30)	0.112
5–10	41 (54.7)	75 (100.0)	1.41 (0.69–2.86)	0.346	0.68 (0.16–2.90)	0.600
≥ 11	19 (44.2)	43 (100.0)	0.92 (0.41–2.08)	0.848	0.50 (0.11–2.29)	0.111
HIV viral load (copies/ml)						
< 1000	70 (37.2)	188 (100.0)	1		1	
≥ 1000	62 (40.3)	132 (100.0)	4.75 (2.69–8.39)	< 0.001	5.58 (2.89–10.78)	< 0.001

*OR* odd ratio, *AOR* adjusted odds ratio, *CI* confidence interval

*Likelihood ratio *p* value age = 0.056

### Factors associated with HPV 16 and/or 18 infections

Table 4 presents both bivariate and multivariable logistic regressions with HPV 16 and/or 18 genotype status as the main dependent variable. In the multivariable regression, number of pregnancies was found to be statistically significant (likelihood ratio *p* value = 0.049). Women who had 1–3 (AOR = 0.09, 95% CI 0.01–0.92, *p* = 0.043), 4–6 (AOR = 0.07, 95% CI 0.01–0.73, *p* = 0.026) and 7 or more pregnancies (AOR = 0.05, 95% CI 0.00–0.65, *p* = 0.022) were respectively 91%, 93% and 95% less likely to have HPV 16 and/or 18 genotype as compared to those who had no pregnancy.

**Table 4:** Predictors of HPV 16 and/or 18 infections among study participants

Variable	HPV 16 and/or 18		OR (95% CI)	*P* value	AOR (95% CI)	*P* value
	Positive	Total				
	n (%)	N (%)				
Age (years)						
25–34	6 (30.0)	20 (100.0)	1		1	
35–44	19 (44.2)	43 (100.0)	1.85 (0.60–5.72)	0.287	3.00 (0.80–11.18)	0.103
45–54	22 (46.8)	47 (100.0)	2.05 (0.67–6.26)	0.206	2.65 (0.69–10.27)	0.158
≥ 55	6 (19.4)	31 (100.0)	0.56 (0.15–2.07)	0.385	0.90 (0.17–4.74)	0.896
Number of pregnancies*						
0	7 (77.8)	9 (100.0)	1		1	
1–3	25 (40.3)	62 (100.0)	0.19 (0.04–1.01)	0.051	0.09 (0.01–0.92)	0.043
4–6	17 (33.3)	51 (100.0)	0.14 (0.03–0.76)	0.023	0.07 (0.01–0.73)	0.026
≥ 7	4 (21.1)	19 (100.0)	0.08 (0.01–0.52)	0.009	0.05 (0.00–0.65)	0.022
HIV Viral load (copies/ml)						
< 1000	29 (41.4)	70 (100.0)	1		1	
≥ 1000	21 (33.9)	62 (100.0)	0.72 (0.36–1.47)	0.372	0.85 (0.38–1.88)	0.683

*Likelihood ratio *p* value for number of pregnancies = 0.049

*OR* odd ratio, *AOR* adjusted odds ratio, *CI* confidence interval

### Factors associated with participants’ knowledge about HPV and cervical cancer

Table 5 summarizes the bivariate and multivariate logistic regressions with women’s knowledge about cervical cancer (insufficient or sufficient knowledge) as the main outcome. In the multivariate model, the educational level of the women was the only variable that remained significant (likelihood ratio *p* < 0.001). Women with primary education were 62% less likely to have sufficient knowledge about cervical cancer as compared to those without any education (AOR = 0.38, 95% CI 0.17–0.82, *p* = 0.014). Women with secondary education had an increased likelihood of having sufficient knowledge of cervical cancer as compared to those without formal education (AOR = 4.15, 95% CI 1.82–9.44, *p* = 0.001).

**Table 5:** Predictors of sufficient cervical cancer knowledge

Variable	OR (95% CI)	*p* value	AOR (95% CI)	*p* value
Age (years)				
25–34	1		1	
35–44	0.72 (0.33–1.57)	0.413	0.91 (0.31–2.72)	0.871
45–54	0.46 (0.21–0.98)	0.043	1.02 (0.29–3.55)	0.981
≥ 55	0.24 (0.11–0.53)	< 0.001	0.39 (0.08–1.79)	0.225
Educational level*				
No education/PRIMARY	1		1	
JHS/secondary	0.40 (0.19–0.83)	0.014	1.82 (0.99–3.32)	0.052
Tertiary	1.14 (0.64–2.02)	0.652	7.55 (1.34–42.43)	0.022
Marital status				
Single	1		1	
Married/cohabiting	0.84 (0.47–1.50)	0.547	0.61 (0.31–1.48)	0.333
Widowed/divorced	0.43 (0.24–0.76)	0.004	0.49 (0.21–1.12)	0.090
Number of pregnancies ever had				
0	1		1	
1–3	0.41 (0.14–1.20)	0.103	0.34 (0.05–2.30)	0.267
4–6	0.34 (0.11–0.99)	0.047	0.34 (0.04–2.67)	0.305
≥ 7	0.18 (0.05–0.60)	0.005	0.24 (0.03–2.36)	0.223
Are you currently sexually active?				
Yes	1		1	
No	0.55 (0.36–0.86)	0.008	1.99 (0.92–4.32)	0.081
Heard of HPV?				
Yes	1		1	
No	0.13 (0.07–0.24)	< 0.001	0.24 (0.11–0.51)	< 0.001
Are you still menstruating?				
Yes	1		1	
No	0.71 (0.19–0.48)	< 0.001	0.22 (0.09–0.54)	0.001
Number of children				
0	1		1	
1–3	0.82 (0.39–1.71)	0.595	3.52 (0.74–16.73)	0.113
4–6	0.48 (0.22–1.04)	0.066	5.16 (0.84–31.67)	0.076
≥ 7	0.18 (0.04–0.77)	0.021	1.88 (0.13–27.62)	0.645
Heard of cervical cancer screening?				
Yes	1		1	
No	0.12 (0.07–0.21)	< 0.001	0.10 (0.05–0.19)	< 0.001

*OR* odds ratio, *AOR* adjusted odds ratio, *CI* confidence interval

*Likelihood ratio *p* value < 0.001

## Discussions

HPV-based cervical cancer screening is recommended globally for both women living with or without HIV. This has implications for LMIC and others with resource limitations, but it also has the potential to address some of the barriers and challenges which have negatively impacted cervical precancer screening efforts in poor resource settings. For WLHIV the need to work toward integrating cervical precancer screening into their routine care cannot be overemphasized. Castle et al. clearly make the case for a comprehensive HIV care and how that would impact not only the attainment of the global HIV elimination targets but also targets for cervical cancer elimination [46]. This study aimed at determining high risk HPV genotype distribution and associated factors among WLHIV and its implication for cervical cancer prevention efforts in a LMIC setting such as Ghana. Using self-collected cervical-vaginal samples, the hr-HPV prevalence was 42.7% with genotypes 59, 18, 35, 58, and 45 being the most common. The prevalence of hr-HPV 16 and/or 18 among the WLHIV was 37.6%.

The socio-demographic characteristics of WLHIV revealed that the mean age of patients was 47.2 years. In relation to cervical precancer screening this would mean that a significant proportion of WLHIV in our setting are outside the WHO’s set priority age group for screening which is 25–45 years [47]. In fact, in our current study, 40.9% of the participants were between 25–44 years, and the remaining were beyond the WHO age priority. Additionally, only a total of 50 out of the 330 WLHIV had ever had cervical screening and for those who had, the main reason (60%) was that they had an opportunity for free screening either as part of a research or an outreach programme followed by request by a clinician as part of diagnostic work up. The above findings compounded by lack of organised screening in the country, the potential cost barrier [35, 48], and the fact that even the health workers do not take advantage of clinic encounters to offer opportunistic screening [49, 50] even among high-risk women explain cervical cancer has a negative effect on women [36, 51, 52]. As found in several studies, most of these WLHIV in LMIC settings like Ghana have low education levels, are married or cohabiting, and are involved in unskilled employment[53, 54]. These and other socio-demographic characteristics have implications for their level of knowledge/awareness as seen in multivariate analysis in this study, their risk perception, and their attitude and practice of cervical screening [37, 53, 55–57]. It is not surprising that less than half of the participants had heard of cervical screening which is consistent with other studies among women in general, WLHIV, and even health care workers in a similar setting as Ghana [27, 49].

The median number of pregnancies among the participants was 4, while 51.2% of the participants had 3–6 lifetime sexual partners, and the mean age at first sex was 18.7 years. Among those who admitted being sexually active within the past 3 months, consistent use of condoms was 77.9%. These are all factors recognised to be associated with the risk of HPV acquisition, and persistence, therefore the risk of cervical cancer [14, 38]. While condoms are known not to be 100% protective against HPV acquisition because of other possible routes of transmission, the level of protection condom offers makes it an important risk reduction strategy [39, 58]. While 39.1% of these WLHIV had ever used hormonal contraceptives, only 16.1% currently use it and consistent with the national picture only 6 women (1.8%) smoke cigarettes. Thus, these factors might not be contributing significantly to the epidemiology of HPV and cervical cancer in the Ghanaian context. In other counties, the proportions and potential impact could be higher [40, 59, 60].

The median duration of living with HIV since confirmed diagnosis was 4 years and the majority (69.1%) of the women had their HIV viral loads < 1000 copies/ml as against 30.9% whose viral loads were ≥ 1000 copies/ml. This study found that WLHIV with higher viral loads had higher odds of having hr-HPV infection. There has been many findings including from a recent systematic review and meta-analysis concerning the role of ART, CD4 count and viral load levels and HPV infection and cervical cancer risk which suggests that sustained viral suppression from ART can have a positive impact on CC prevention [18, 19, 41, 42, 61].

Our current hr-HPV prevalence (43.7%) is lower than what we reported in a previous study (65.6%) that relied on another testing method (Anyplex-II HPV 28) and involved a cohort of WLHIV who had more evidence of immunosuppression [12]. It is worth noting that 69.1% of the WLHIV in the current study had viral loads < 1000 copies/ml. A recent systematic review by Bogale et al. reported pooled hr-HPV prevalence of 51% which is higher than found in this study [61]. The high prevalence of hr-HPV among such WLHIV has implications for screening algorithms used in especially LMIC settings. Hence, the adoption of a screen-triage-treat approach might be more manageable, it requires a carefully selected triage approach to ensure sensitivity and specificity are improved [62, 63]. Countries like Ghana, explore various HPV-based testing platforms based on their performance and their suitability for the peculiar resource limitations [64]. Duan et al. in their study among WLHIV in China recommended the use of HPV testing with restricted genotyping using self-collected samples followed by triaging as used in this study [65]. Desai et al. also evaluated using self-collection of samples for HPV testing followed by Colposcopy for triaging for treatment and proposed a possible role of partial genotyping [66]. The essence of these studies is for LMICs to find the best algorithm in terms of cost-effectiveness, and acceptability among others to inform national screening guidelines.

Inasmuch as the algorithm used for this study conforms to WHO recommendations, its use in a LMIC like Ghana, is fraught with numerous challenges. The first amongst them is the health illiteracy level of the population, which fosters people’s desire to dwell on cultural and religious beliefs and misconceptions and negatively influence the assimilation of cervical cancer prevention education and the utilization of available services. The second challenge is the existence of scanty number of facilities with structured cervical cancer prevention programmes, which is a reflection of the non-existence of national programmes in many LMICs like Ghana. As a result, existing prevention programmes are largely opportunistic and unsustainable. The limited number of laboratories, capable of performing HPV DNA test, pose a big challenge to accessibility; there are no more than four of such facilities in Ghana and these are mostly centered in the national Capital. Coupled with this is the cost of testing, which is beyond the reach of many. Lastly, in an environment, where there is no proper address system and poverty is rife, the proportion of loss to follow-up is great, as client-tracing becomes an arduous task and many clients, who need to make visits for follow-up are unable to do so due to financial constraints. These challenges, among others, pose a serious threat to the quest to roll out effective, comprehensive and sustainable cervical cancer prevention programmes in LMIC like Ghana.

Concerning genotypes, hr-HPVtypes 52 and 16 are not as prevalent in this study as in our previous study though even in this study they still feature prominently beyond the top 5 common hr-HPVs [12]. In this study hr-HPV 59 is the commonest hr-HPV found and hr-HPV 45 also appears as one of the top 5 h-HPVs. These findings are in line with other studies that have reported hr-HPV 59 and 45 among the commonest hr-HPV found in cervical cancer samples in Ghana. [13]. In addition, hr-HPV 35 and 45 have been reported to be among the commonest hr-HPV types in a number of studies from the Sub-Saharan African Region and both are among the most common in this study [8, 43, 67] Though hr-HPV-16 is not among the most common hr-HPVs, 37.6% of patients were infected with both hr-HPV 16/18, both of which are also reported to be among the common hr-HPVs detected in cervical cancer samples studied in Ghana [13, 43]. The reported higher risk of progression to cervical cancer associated with types 16 and 18, forms a basis for the role even partial genotyping which focuses on identifying these can play in CC screening programmes. In our setting, there is the need to evaluate the utility of partial genotyping for screening in the context of the commonest hr-HPV genotypes.

Though phylogenetically linked to other common hr-HPVs, the high prevalence of hr-HPV types such as 35, and 59 among others may warrant their inclusion in subsequent vaccines for our region of the world [44, 68, 69]. This may make the vaccines more efficacious in our population. In this study 66% of women have hr-HPV types covered by the current nonavalent vaccine and similar high coverage was found by Ndizeye et al. in Burundi [70] and yet this vaccine is generally unavailable even at a personal cost in Ghana and many African countries. In general, the regional/geographical variation in hr-HPV types is underscored by the differences in hr-HPV types reported for different regions of the world. It is, therefore, essential for HPV vaccination efforts across Africa for females, particularly those of higher-risk groups,to be strengthened. This has been demonstrated to be effective in the LMIC setting, hence the need to have a strategic plann for such countries keeping the cost implication and other factors in mind [71–74].

## Limitation

This study was a single facility-based cross -sectional study and we recognise that larger national studies among WLHIV are needed to guide the national guidelines. While acknowledging this limitation, we believe that it has contributed to the growing body of evidence that supports the use of a self-collected HPV-based screen-triage-treat approach, as part of comprehensive HIV care in settings like Ghana.

## Conclusion

The prevalence of hr-HPV among WLHIVremains high in this study with high rates of multiple infections and infection with genotypes 16and/or18. An association was found between HIV viral load and hr-HPV infection. The study also identified knowledge/awareness gap and other potential areas for intervention to reduce the incidence of cervical cancer among WLHIV in Ghana, such as increasing awareness of cervical cancer, the possible role of vaccination, screening and follow up.

The study also suggests that an HPV-based screen-triage-treat approach with partial genotyping must be considered as part of national programmes for LMICs like Ghana. Comprehensive HIV care for these women should include education on cervical cancer and its prevention.

## Data Availability

All data relevant to this manuscript have been included in the results section. The dataset could be requested from the corresponding author upon reasonable request.

## Abbreviations

AIS: Adenocarcinoma in situ
AIDS: Acquired immunodeficiency syndrome
AOR: Adjusted odds ratio
ART: Anti-retroviral therapy
CAPI: Computer assisted personal interviewing
CC: Cervical cancer
CCTH: Cape Coast teaching hospital
CI: Confidence interval
CIN: Cervical intra-epithelial neoplasm
DNA: Deoxyribonucleic acid
HIV: Human immunodeficiency virus
HPV: Human papillomavirus
Hr-HPV: High risk human papillomavirus
ICC: Invasive cervical carcinoma
IQR: Interquartile range
LEEP: Loop electrosurgical incision procedure
LMIC: Low middle-income country
OB/GYN: Obstetrics and gynaecology
OR: Odds ratio
PAP: Papanicolaou
SD: Standard deviation
SIL: Squamous intraepithelial lesions
STIs: Sexually transmitted infections
VIA: Visual inspection with acetic acid
WLHIV: Women living with HIV
